# Advances in epigenetic therapeutics with focus on solid tumors

**DOI:** 10.1186/s13148-021-01069-7

**Published:** 2021-04-20

**Authors:** Ning Jin, Tiffany L. George, Gregory A. Otterson, Claire Verschraegen, Haitao Wen, David Carbone, James Herman, Erin M. Bertino, Kai He

**Affiliations:** 1 The Ohio State University Comprehensive Cancer Center – Arthur G. James Cancer Hospital and Richard J. Solove Research Institute, Columbus, OH USA; 2 Department of Microbial Infection and Immunity, The Ohio State University, Columbus, OH USA; 3 Department of Medicine, UPMC Hillman Cancer Center, University of Pittsburgh School of Medicine, Pittsburgh, PA USA

**Keywords:** Epigenetic, Therapeutics, Therapies, Cancer, Methylation, Acetylation, Reprogramming

## Abstract

Epigenetic (“above genetics”) modifications can alter the gene expression without altering the DNA sequence. Aberrant epigenetic regulations in cancer include DNA methylation, histone methylation, histone acetylation, non-coding RNA, and mRNA methylation. Epigenetic-targeted agents have demonstrated clinical activities in hematological malignancies and therapeutic potential in solid tumors. In this review, we describe mechanisms of various epigenetic modifications, discuss the Food and Drug Administration-approved epigenetic agents, and focus on the current clinical investigations of novel epigenetic monotherapies and combination therapies in solid tumors.

## Background

Carcinogenesis is a complex process that involves both genetic and epigenetic changes, leading to the transformation of normal cells into malignant cells. The aberrant genetic and epigenetic alterations are the hallmark of cancer. Epigenetic modifications are responsible for cellular plasticity, differentiation and reprogramming without altering the underlying DNA sequence of the organism [1]. Normal cell development depends on regulated transcription of critical proteins, and individual cells within specific tissues and organs maintain their unique biological functions based on heritable and evolutionary differences in the DNA packaging. Histone proteins (two copies of histones H2A, H2B, H3 and H4) wrap around 147 base pairs of DNA to form a nucleosome. Nucleosomes are further compacted by additional proteins to form chromatin. Epigenetic modifications, including acetylation and methylation (histone marks), can alter DNA accessibility and chromatin structure and regulate gene transcription activation or silencing. Acetylated histones are less compact, thereby enabling gene transcription by making the DNA more accessible to RNA polymerase and the transcriptional machinery. On the other end, methylated histones can be either repressive or activating, depending on the site and degree of methylation. Methylation of histone H3 at lysine 4, 36 and 79 is generally considered as an activation mark, whereas methylations on histone H3 lysine 9, 27 are linked to transcriptional repression [2]. In general, enzymes that add acetyl or methyl groups to the histone or DNA are referred to as “writers”, whereas enzymes that remove histone marks are called “erasers”. Proteins that recognize histone and DNA modifications are the chromatin “readers” [1].

**Fig. 1:**
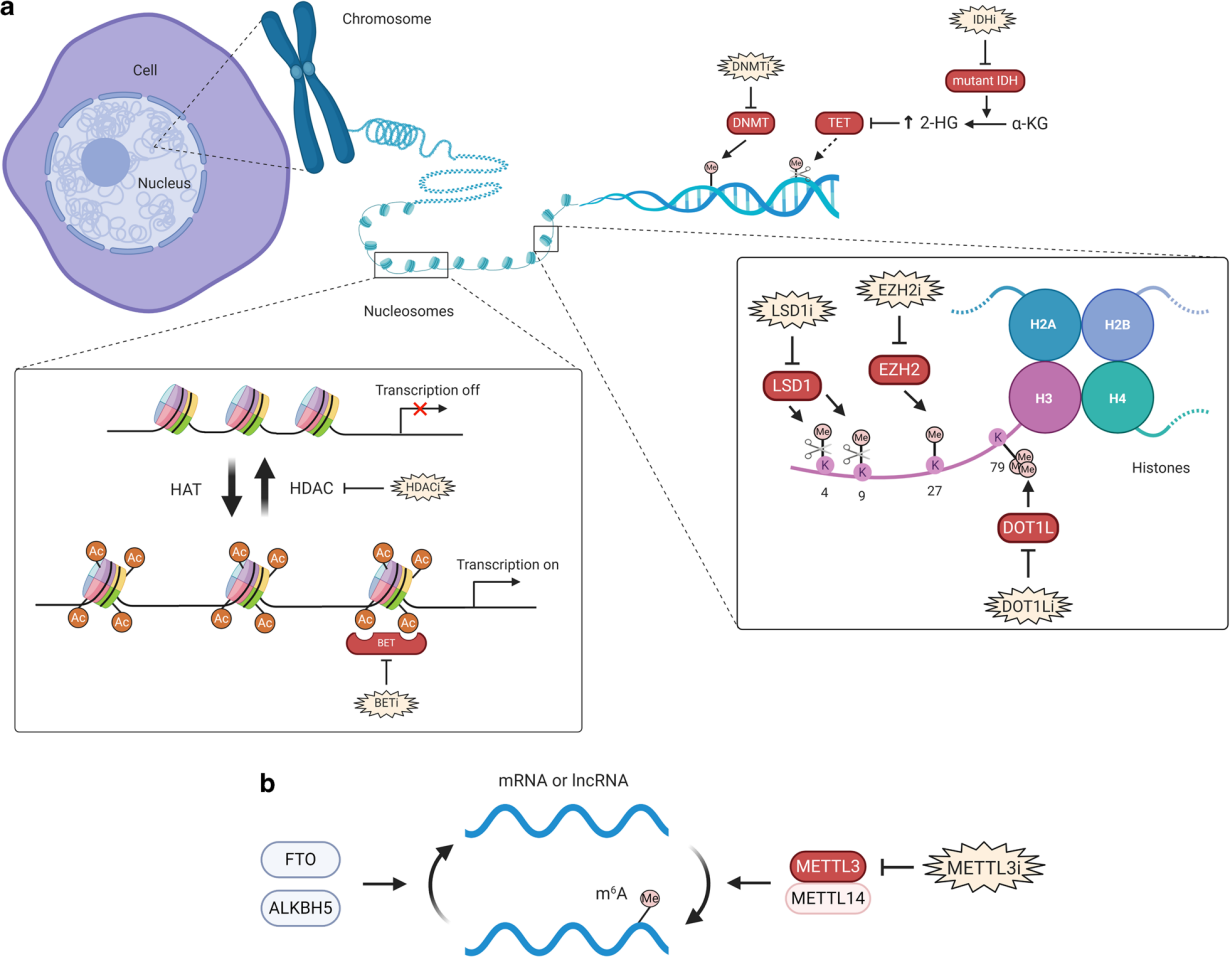
The epigenetic readers, writers and erasers. (**a**) Histone proteins wrap around DNA to form a nucleosome, which are then compacted to form chromatins and further into chromosomes. HATs add acetyl groups and HDACs remove acetyl groups from histone lysine residues. The acetylated histones are considered as “open chromatin”, enabling gene transcription, whereas deacetylated histones are “closed chromatin” and associated with gene silencing. BET proteins recognize acetylated histones and are involved with transcriptional activation by recruiting other proteins. In comparison with histone acetylation, histone methylation can be either repressive or activating, depending on the site and degree of methylation. Different histone methyltransferases are specific to modify the lysine or arginine residues. LSD1 demethylates either the active mark of H3K4 or the repressive mark of H3K9, in a context-dependent manner. EZH2 methylates H3K27 and promotes transcription silencing. DOT1L methylates H3K79, which is an activation mark. At the DNA level, DNMTs methylate and convert cytosine to 5-methylcytosine (5mC), and TETs remove methyl groups on DNA. Mutations in genes encoding enzymes in the cellular metabolism can alter the epigenetic landscape. This is exemplified by IDH1/2 that metabolize isocitrate to α-KG. IDH1/2 mutations (gain-of-function) result in further processing of α-KG to 2-HG (“oncometabolite”), which inhibits TETs and leads to reduced DNA demethylation (increased DNA methylation state). **b** A multiprotein complex (consisting METTL3, METTL14 and other subunits) methylates adenosine base at the nitrogen-6 position and forms m^6^A in the messenger RNA. m^6^A modification is reversible and it can be erased by ALKBH5 and FTO. m^6^A reader proteins can regulate the metabolism of mRNA. For example, YTHDF2 binds to m^6^A and targets mRNA degradation. *HAT* histone acetyltransferase, *HDAC* histone deacetylase, *BET* bromodomain and extra-terminal motif proteins, *LSD1* lysine-specific histone demethylase 1, *EZH2* enhancer of zeste homolog 2, *DOT1L* disruptor of telomeric silencing 1 like, *DNMT* DNA methyltransferase, *TET* ten-eleven translocation, *IDH* isocitrate dehydrogenase, *α-KG* α-ketoglutarate, *2-HG* 2-hydroxyglutarate, *m*^*6*^*A* N^6^-methyladenosine, *METTL3* methyltransferase-like protein 3, *METTL14* methyltransferase-like protein 14, *ALKBH5* alkB homolog 5, *FTO* fat-mass and obesity associated protein

The complex balance of normal and abnormal epigenetic regulation is an area of intense interest in cancer research, including therapeutic development in cancer [3]. This article will illustrate aberrant changes in DNA methylation, histone acetylation and histone methylation (summarized in Fig. 1) in cancer, discuss the epigenetic agents in both hematological malignancies and solid tumors, and highlight the recent novel combination strategies, such as with immune checkpoint inhibitors and hormonal therapies, in solid tumors.

## Main text

### Therapeutics targeting the cancer epigenome

Therapeutics targeting the cancer epigenome can be grouped into two major categories: broad spectrum reprogrammers and narrowed spectrum reprogrammers [4]. An argument can be made for the potential effectiveness of both broad and targeted epigenetic therapies. Broad-spectrum reprogrammers include the inhibitors of DNA methyltransferase (DNMT), histone deacetylase (HDAC) and the bromodomain and extra-terminal motif proteins (BETs). These drugs cause genome-wide cancer-specific gene expression alterations. In contrast, narrowed spectrum epigenetic modifying agents targeting lysine-specific histone demethylase 1 (LSD1), enhancer of zeste homolog 2 (EZH2), DOT1-like histone lysine methyltransferase (DOT1L), to achieve precise inhibition of epigenetic regulatory proteins.

### Broad spectrum reprogrammers

#### DNMT (DNA methyltransferase—“writer”) inhibitors

DNA methylation affects the transcription of genes without altering the DNA sequence. In eukaryotic DNA, cytosine is methylated and then converted into 5-methylcytosine by DNMTs [5]. Hypermethylation of specific regions, such as the CpG islands of tumor suppressor genes, plays an important role in carcinogenesis for many types of cancers [6–8]. There are 3 primary DNMTs—DNMT1, DNMT3A and DNMT3B [9–11]. DNMT1 is predominantly involved in maintaining the preexistent methylation pattern during DNA replication. DNMT3A and DNMT3B are involved in facilitating de novo DNA methylations at loci that were previously unmethylated [12]. Tumorigenesis often involves an interplay among all 3 DNMTs [13–16]. DNMT inhibitors act as cytidine analogs and induce loss of DNA methylation. There are two main classes of hypomethylating agents, the nucleoside analogs (such as 5-azacitidine that incorporates into DNA and RNA and 5-aza-2′-deoxycytidine, or decitabine, that incorporates into DNA) and the anti-sense DNA methyltransferase inhibitors (such as MG98) that do not require incorporation into DNA. The ability of azacitidine to be incorporated into DNA and RNA can lead to broad biological effects in resting and dividing cells [17]. DNMT inhibitors have shown to be particularly effective in targeting DNA methylation in leukemic cells [18, 19].

#### HDAC (histone deacetylase—“eraser”) inhibitors

Histone modification occurs via acetylation of lysine residues. Two families of enzymes, histone acetyltransferases (HATs) and histone deacetylases (HDACs), operate in an opposing manner. HATs acetylate lysines within the amino-terminal tails of histone proteins, resulting in relaxation of chromatin structure and facilitating gene activation. Conversely, HDACs remove acetyl groups from hyperacetylated histones and make the chromatin condensed and transcriptionally silent. There are four classes of HDAC enzymes based on their structures and functions: class I (HDAC 1–3 and 8), IIa (HDAC 4, 5, 7, 9), IIb (HDAC 6, 10), III (Sir-2 related—SIRT1-7) and IV (HDAC 11) [20, 21]. Class I HDAC proteins are mainly localized in the nucleus, whereas class II HDACs are expressed in a more tissue-restricted manner [22]. Sharing significant homology with both Class I and Class II HDACs, class IV HDAC does not possess a nuclear localization signal and its function is largely unknown [23]. HDACs are key elements in the regulation of gene expression, differentiation and development, and the maintenance of cellular homeostasis. HDAC inhibition causes global gene upregulation (potential oncosuppressors) and leads to arrest of tumor cell growth, apoptosis and anti-angiogenesis [24, 25]. In addition, HDAC facilitates the binding of elongation factors to acetylated promoters and enhancers for efficient elongation. Therefore, HDAC inhibitors block gene elongation and inhibit gene expression, especially in highly expressed genes (oncogenes) [26]. Many HDAC inhibitors are non-specific and can be used to inhibit multiple isoforms of HDACs.

#### BET (bromodomain and extra-terminal motif proteins—“reader”) inhibitors

BET proteins are known to recognize acetylated lysine in chromatin [27]. The BET family of proteins include BRD2, BRD3, BRD4, and the testes-specific BRDT [28, 29]. Bromodomains can specifically bind acetylated lysine residues of histone proteins, and are involved with histone modifications, chromatin remodeling and transcriptional activation via recruitment of other proteins [30, 31]. BRD2 and BRD3 facilitate the passage of RNA Pol II to elongate the DNA transcripts through hyperacetylated nucleosomes [32]. BRD4 enhances the recruitment of positive transcription elongation factor b (P-TEFb), leading to the release of Pol II from a pause in transcription elongation in the promoter-proximal region [33]. In particular, aberrant BRD4 expression contributes to carcinogenesis by mediating hyperacetylation of the chromatin associated with cell proliferation-promoting genes [34]. Suppression of BRD4 led to anti-leukemic effects in acute myeloid leukemia (AML) mouse models and revealed a potential epigenetic target for AML [35]. In addition, BRD4 and BET proteins also regulate enhancer (a short region of DNA that can be bound by transcription factors to enhance the transcription of a particular gene) function and, in particular, large clusters of enhancers (super-enhancers), which drive oncogene expression, such as BCL-2 and c-MYC [36, 37]. Interestingly, the pathogenic fusion product of NUT (nuclear protein in testis) with BRD4 or BRD3 (BRD4-NUT or BRD3-NUT) causes NUT midline carcinoma (NMC), which is a rare but poorly differentiated and highly aggressive cancer of the squamous cell lineage that arises in midline structures [38]. BET bromodomain blockade using small-molecule inhibitors leads to selective repression of the transcriptional network driven by c-MYC [39].

#### METTL3 (methyltransferase like-3—“writer”) inhibitors

In addition to the epigenetic modifications on either DNA or histones, methylation is also observed in eukaryotic RNAs, including messenger RNA (mRNA), microRNA (miRNA) and long non-coding RNA (lncRNA), etc. Methylation modification impacts RNA processing, nuclear export, translation initiation and degradation [40]. In particular, N^6^-methyladenosine (m^6^A) modification of mRNA is most abundant, which occurs in two consensus sequence motifs including G(m^6^A)C primarily and A(m^6^A)C to a lesser extent [41, 42]. m^6^A is installed by a multiprotein writer complex that consists of methyltransferase-like protein 3 (METTL3), methyltransferase-like protein 14 (METTL14) and other accessory subunits. m^6^A modification is reversible and it can be erased by ALKBH5 (alkB homolog 5) [43] and FTO (fat-mass and obesity associated protein) proteins (Fig. 1) [44]. In addition, METTL3 and METTL14 are also identified as key actors of adenosine methylation of miRNAs [45, 46], whereas FTO is recognized as a key actor of adenosine demethylation of miRNAs [47]. m^6^A reader proteins can specifically bind to m^6^A transcripts and regulate the metabolism of mRNA [48]. For example, YTHDF2 (YTH domain family 2) binds to m^6^A in mRNA and targets mRNA degradation, whereas YTHDF1, YTHDF3, and eukaryotic initiation factor 3 (eIF3) promote translation of mRNA transcripts [49]. METTL3 has been found to be upregulated with increased m^6^A levels in cancer compared with those in normal tissues, suggesting a potential oncogenic role in different cancer types including AML, renal cell carcinoma, non-small cell lung cancer (NSCLC) and gastric cancer [50–53]. The studies show that loss of either METTL14 or METTL3 in AML cell lines and primary leukemic blasts led to induction of differentiation [50, 54]. In addition, METTL3 has been associated with multiple cell signaling pathways, including tumorigenesis, proliferation, invasion, migration, cell cycle, differentiation and cell viability [55]. Currently, multiple METTL3 inhibitors are under investigation in both AML and solid tumors, with pending clinical trials in the near future [56].

Besides the role of METTL3 in m^6^A modification on mRNAs and miRNAs, recent study suggested that DNMT3A methylates miRNA at cytosine residues and inhibits the formation of miRNA/mRNA duplex, leading to the loss of their repressive function in gene expression [57]. Therefore, using demethylating agent to block miRNA methylation may broaden its therapeutic potentials.

### Narrowed spectrum reprogrammers

#### LSD1 (histone demethylase—“eraser”) inhibitors

LSD1 (lysine-specific histone demethylase 1, also known as KDM1A) is the first discovered histone lysine demethylase with the ability to erase the mono-methyl and di-methyl chromatin marks on histone H3, predominantly at lysines 4 and 9 (H3K4 and H3K9) [58–60]. It can also demethylate non-histone proteins, including DNMT1 and TP53 [59]. Moreover, LSD1 is a multifunctional subunit of both repressive and activating histone-modifying complexes and can therefore act as both a transcriptional repressor or activator in a context-dependent manner [61]. LSD1 regulates the balance between self-renewal and differentiation of stem cells, and LSD1 inhibition in mixed lineage leukemia (MLL)-rearranged leukemia has been shown to downregulate expression of some leukemia associated genes and cause apoptosis and cell differentiation [62]. In addition, LSD1 is overexpressed in various solid tumors including prostate, breast, lung and colorectal cancers, and neuroblastoma [63–67]. Pharmacological inhibition of LSD1 leads to inhibition of proliferation, differentiation, invasion, and migration in vitro and in vivo [68]. Thus, LSD1 inhibitors might be promising potential therapeutic options in a variety of cancers. Recently, it has been demonstrated that the effects of LSD1 inhibitors are particularly robust for small cell lung cancer (SCLC) through promotion of differentiation of tumor-enriched stem-like cells [69].

#### EZH2 (histone methyltransferase—“writer”) inhibitors

Several families of histone methyltransferases (HMT) that catalyze the methylation of specific lysine residues in histones H3 and H4 have been identified [70]. Unlike other histone modifications, which simply specify active or repressed chromatin states, histone lysine methylations confer active or repressive transcription depending on their positions and methylation states [71]. EZH2 (enhancer of zeste homolog 2), a histone methyltransferase and a catalytic component of polycomb repressive complex 2 (PRC2), catalyzes tri-methylation of histone H3 at lysine 27 (H3K27me3) to promote transcription silencing [72, 73]. Through modulating critical gene expression, EZH2 promotes cell survival, proliferation, epithelial-to-mesenchymal transition (EMT), invasion, and drug resistance of cancer cells [74]. EZH2 is activated by mutations (gain-of-function) in lymphoma [75], and EZH2 overexpression is associated with aggressiveness and worse clinical outcome in several solid tumors, including prostate, breast, bladder, and endometrial cancers, and melanoma [76–78]. The use of an EZH2 inhibitor demonstrated selective killing effect in cell lines carrying EZH2 activating mutations [79]. Several studies also identified a PRC2-independent function of EZH2 in transcriptional activation, involving transcription of androgen receptor (AR), estrogen receptor (ER) and Wnt signaling [80–83]).

#### DOT1L (histone methyltransferase—“writer”) inhibitors

Disruptor of telomeric silencing 1 (DOT1) is a novel class of HMT that was first identified to dysregulate gene silencing near telomeres in yeast [84]. DOT1-like (DOT1L) is the only known methyltransferase that deposits mono-, di-, and trimethyl marks on histone H3 lysine 79 (H3K79) in mammals. It participates in the regulation of transcription, differentiation and proliferation of normal cells. DOT1L has been shown to be critical for transformation by MLL fusion proteins in AML [85, 86]. Preclinical models demonstrate that MLL-driven leukemia is particularly sensitive to inhibition of DOT1L activity, and DOT1L inhibitors have been shown to specifically reduce H3K79 methylation marks and expression of MLL-fusions target genes in leukemic cells [87]. In addition, a recent study demonstrated the role of DOT1L in breast cancers that do not harbor a MLL translocation. DOT1L plays an important role in the initiation and progression of breast cancer by targeting the gene expression of EMT-promoting factors, suggesting DOT1L to be a therapeutic target for aggressive breast cancer [88]. While the pre-clinical studies showed promising activity of DOT1L inhibitors, the phase I study of DOTlL inhibitor, pinometostat, in adult and pediatric patients with relapsed or refractory leukemia demonstrated limited clinical response [89, 90].

#### IDH (isocitrate dehydrogenase) inhibitors

Mutations in genes encoding enzymes of the tricarboxylic acid (TCA) cycle can disrupt cell metabolism and alter the epigenetic landscape. For example, IDH1/2 enzymes metabolize isocitrate to α-ketoglutarate (α-KG) in the TCA cycle. α-KG serves as a co-factor for α-KG-dependent dioxygenases, including the ten-eleven translocation (TET) family of DNA demethylases and Jumonji family of histone demethylases. TET family of DNA methylases act on methylated DNA sequences, convert 5-methylcytosine (5mC) to 5-hydroxymethylcytosine (5hmC), 5-formylcytosine (5fC) and 5-carboxylcytosine (5caC), which will ultimately remove methyl groups and ensure the correct DNA methylation in the cell [91]. IDH1/2 mutations are found in several cancer types, including AML, gliomas, chondrosarcoma and intrahepatic cholangiocarcinoma [92, 93]. IDH mutations (gain-of-function) result in further processing of α-KG to 2-hydroxyglutarate (2-HG). This leads to the production of “oncometabolite” 2-HG, which inhibits TET family of DNA demethylases and Jumonji family of histone demethylases [94] and promotes tumorigenesis [95]. Accumulation of 2-HG in leukemic cells leads to increased DNA and histone methylation and results in blocked cell differentiation [96, 97]. Several small molecule inhibitors of both IDH1 and IDH2 have demonstrated reduction of 2-HG levels and differentiation of leukemic cells that carry the specific IDH mutations [98–100]. These effects also correlate with global changes in DNA methylation/histone modification state, suggesting that the phenotypic effects are, to some extent, secondary to rewiring transcriptional programs in the leukemic cells [101].

The aforementioned RNA demethylases, FTO and ALKBH5 which demethylate m^6^A, are α-KG-dependent dioxygenases [102–104]. m^6^A destabilizes transcripts and controls expression of key transcription factors in hematopoietic stem cells (HSCs) and human embryonic stem cells (ESCs) [105]. 2-HG suppresses FTO activity in leukemia cells, leading to decreased expression of the lineage transcription factor CCAAT enhancer binding protein α (C/EBPα) that enforces normal HSC quiescence and myeloid differentiation [106]. Therefore, the inhibition of IDH may lead to the changes in metabolic activities in TCA cycle such as α-KG and 2-HG, coordinating the cell fate in HSCs and ESCs.

### Epigenetic drugs for cancer treatment: approved or in clinical trials

#### Approved epigenetic therapies

To date, the FDA-approved epigenetic agents are mostly limited in treating hematologic malignancies. Two DNMT inhibitors are approved for the treatment of myelodysplastic syndrome (MDS)—azacitidine and decitabine. Clinical trials with azacitidine and its deoxy derivative, decitabine, demonstrated that 15% or more of the patients with AML or intermediate to high-risk MDS showed improvement in blood cell counts and survival [107, 108]. Several HDAC inhibitors are approved for the treatment of hematologic malignancies, including belinostat for peripheral T cell lymphoma (PTCL), panobinostat for multiple myeloma, vorinostat for cutaneous T cell lymphoma (CTCL) and romidepsin for both CTCL and PTCL. IDH inhibitors, enasidenib and ivosidenib, have been approved for relapsed or refractory AML with IDH mutations [109–111]. EZH2 inhibitor, Tazemetostat, has been approved for patients with relapsed or refractory follicular lymphoma (R/R FL) with EZH2 mutation and who have received at least 2 prior systemic therapies, and for adult patients with R/R FL who have no satisfactory alternative treatment options [112].

Clinical trials are ongoing in solid tumors with agents from multiple drug classes. In January 2020, tazemetostat has been granted accelerated approval by FDA in treating epithelioid sarcoma, for which we will discuss later in this article [113]. These FDA-approved agents are summarized in Table 1.

**Table 1:** FDA-approved epigenetic therapeutics in malignancies

Epigenetic therapeutics	Target	Date of approval	Approved indication	Reference
*DNMTi*				
Azacitidine (Vidaza)	DNMT-1 inhibition	5/2004	MDS	[172–174]
Decitabine (Dacogen)	DNMT-1 inhibition	5/2006	MDS	[175]
*HDACi*				
Vorinostat (Zolinza)	Class I and II HDACs	10/2006	Progressive, persistent, or recurrent CTCL disease on or following two systemic therapies	[176, 177]
Romidepsin (Istodax)	Class I HDACs primarily	11/2009	CTCL after at least one prior systemic therapy	[178, 179]
		5/2011	PTCL after at least one prior therapy	
Belinostat (Beleodaq)	Class I, II and IV HDACs	7/2014	Relapsed or refractory PTCL	[180]
Panobinostat (Farydak)	Class I, II and IV HDACs	2/2015	MM (in combination with bortezomib and dexamethasone) after at least two prior regimens, including bortezomib and an immunomodulatory agent	[181]
*IDH mutation inhibitor*				
Enasidenib (Idhifa)	IDH2 mutant enzyme	8/2017	Relapsed or refractory AML with an IDH2 mutation	[109]
Ivosidenib (Tibsovo)	IDH1 mutant enzyme	7/2018	Relapsed or refractory AML with an IDH1 mutation	[110, 111]
*EZH2 inhibitor*				
Tazemetostat (Tazverik)	EZH2 inhibition	6/2020	Relapsed or refractory (R/R) FL with EZH2 mutation and who have received at least 2 prior systemic therapies, and for adult patients with R/R FL who have no satisfactory alternative treatment options	[113]
		1/2020	Metastatic or locally advanced epithelioid sarcoma not eligible for complete resection	[112]

*CTCL* cutaneous T-Cell lymphoma, *DNMT-1* DNA demethyltransferase-1, *DNMTi* DNA methyltransferase inhibitor, *FDA* US Food and Drug Administration, *FL* follicular lymphoma, *HDACi* histone deacetylase inhibitor, *IDH* isocitrate dehydrogenase, *MDS* myelodysplastic syndrome, *MM* multiple myeloma, *PTCL* peripheral T-cell lymphoma, *CTCL* cutaneous T-cell lymphoma

#### Monotherapies in solid tumors

Historically, the first generation DNMT inhibitors (azacytidine and decitabine) showed limited activity in solid tumor, in part due to their toxicity. Biomarker studies demonstrated evidence of DNA methylation changes associated with drug administration; however, the responses were short-lived and treatment resistance developed early [114–117]. A phase I study of decitabine was conducted in patients with stage IV lung cancer, esophageal cancer, and malignant pleural mesothelioma. No objective response was observed and severe toxicities occurred. Grade 4 neutropenia was observed in 43% (15 out of 35) of the patients and grade 3 hepatotoxicity were seen in two patients with extensive liver metastases [118].

The second-generation DNMT inhibitors, such as guadecitabine (SGI-110), have been undergoing investigation. Guadecitabine is a novel hypomethylating prodrug of decitabine with a prolonged half-life. This novel compound is an oligonucleotide consisting of decitabine linked through a phosphodiester bond to the endogenous nucleoside deoxyguanosine. The dinucleotide configuration provides protection from drug clearance [119]. Guadecitabine [119] has been demonstrated to be safe and well tolerated as a single agent, with evidence of promising activity in heavily pretreated MDS and AML patients [120]. A phase II trial of SGI-110 monotherapy in patients with HCC who progressed on sorafenib (NCT01752933) was completed. The single agent SGI-110 demonstrated disappointing PFS in this trial.

Similar to DNMT inhibitors, HDAC inhibitors have shown limited single agent activity, and responses have been rare in solid tumors [121, 122]. A phase II study of vorinostat in relapsed non-small cell lung cancer (NSCLC) showed no objective response in 14 evaluable patients, and severe toxicities were reported including neutropenia, lymphopenia, fatigue and pulmonary embolisms [123]. A phase III trial of vorinostat as second-line monotherapy in advanced mesothelioma was conducted in patients who had previously received chemotherapy, and it showed that single agent vorinostat did not improve overall survival (OS) compared with placebo [124]. Representative recent clinical trials of single agent DNMT inhibitors and HDAC inhibitors in solid tumors are summarized in Table 2.

**Table 2:** Clinical trials of single agent DNMT inhibitors and HDAC inhibitors in solid tumors

Agent(s)	Cancer type(s)	Trial details	Trial identifier/status
*DNMT inhibitors*			
CC-486 (oral form of azacitidine)	Locally advanced or metastatic NPC	Phase II trial	NCT02269943
		Enrollment: 36 patients	Completed 4/2017
		Results: ORR 12%; median PFS and OS were 4.7 and 18.0 months, respectively. CC-486 as monotherapy did not show sufficient clinical activity in this patient population. The most common grade 3/4 TEAEs were neutropenia (33%) and febrile neutropenia (11%) [182]	
Guadecitabine (SGI-110)	Advanced HCC	Phase II trial	NCT01752933
		Enrollment: 52 patients	Completed 9/2015
		Results: DCR 25% and 24.4%, median duration of response 262 days and 144 days, median PFS 55 days and 82.5 days, median OS 294 days and 245 days in the 60 mg/m2 group and 45 mg/m2 group, respectively. The most common being febrile neutropenia in both groups (25% vs. 11%) [183]	
ASTX727 (cedazuridine and decitabine)	Recurrent or progressive non-enhancing IDH mutant gliomas	Phase I trial	NCT03922555
		Enrollment: 18 patients	Recruiting
		Results: pending	
*HDAC Inhibitors*			
Entinostat (SNDX-275, MS-275)	Relapsed or refractory abdominal neuroendocrine tumors	Phase II trial	NCT03211988
		Planned enrollment: 40 patients	Recruiting
		Results: N/A	
Mocetinostat (MGCD0103)	Locally advanced or metastatic urothelial carcinoma	Phase II trial	NCT02236195
		Enrollment: 17 patients	Completed 7/2016
		Results: Eligible patients received oral mocetinostat at a dose of 70 mg thrice weekly (TIW) escalating to 90 mg TIW in 28-day cycles in a planned 3-stage study. Single agent mocetinostat was not efficacious in this setting and significant toxicities impacted drug exposure and possibly contributed to modest clinical activity in these pretreated patients [184]	
Panobinostat (LBH589)	Locally recurrent or metastatic HER2-negative breast cancer	Phase II trial	NCT00777049
		Enrollment: 54 patients	Completed 4/2015
		Results: In HR + group (n = 33) there were 1 PR, 13 SD, 14 PD and 5 missing data; most common SAE was thrombocytopenia (12.5%). In HR-group (n = 21) there was 1 CR, 4 SD, 14 PD, 2 missing data; most common SAE was constipation (10%)	
	Metastatic medullary thyroid cancer and radioactive iodine resistant differentiated thyroid cancer	Phase II trial	NCT01013597
		Enrollment: 13 patients	Completed 2/2016
		Results: Patients received LBH589 20 mg by mouth three times weekly for 28-day cycles. No responses seen, median time to progression 3.6 months, median OS 18.4 months (5.8 to NA). Most common toxicities were lymphopenia, thrombocytopenia and fatigue (8 patients each). There were 3 deaths “not otherwise specified”	
	Metastatic melanoma	Phase I trial	NCT01065467
		Enrollment: 16 patients	Completed 3/2017
		Results: 6 patients were treated on Arm A (oral panobinostat 30 mg daily on MWF) and 10 patients were enrolled to Arm B (oral panobinostat 30 mg three times a week every other week) with 9 patients treated. DLT in arm A included clinically significant thrombocytopenia requiring dose interruption. Among all 15 treated patients, ORR was 0% and DCR was 27%. Panobinostat monotherapy was not active in melanoma and there was a high toxicity rate [185]	
Valproic acid (VPA)	Uveal melanoma	Phase II trial	NCT02068586
		Planned enrollment: 150 patients	Recruiting
		Results: N/A	
	Advanced thyroid cancers of follicular origin	Phase II trial	NCT01182285
		Enrollment: 13 patients	Completed 4/2016
		Results: No responses were seen and 6 patients had PD. Zero of 10 patients had increased radioiodine uptake at their tumor sites. Valproic acid did not increase radioiodine uptake and did not have anticancer activity in patients with advanced, radioiodine-negative thyroid cancer of follicular cell origin [186]	
Vorinostat (SAHA)	Locally advanced, recurrent or metastatic adenoid cystic carcinoma	Phase II trial	NCT01175980
		Enrollment: 30 patients	Completed 6/2018
		Results: Stable disease was the best response in 27 patients. Median PFS and stable disease duration were both 11.4 months and median OS has not been reached. Grade 3 AEs that occurred in more than 1 patient included lymphopenia (n = 5), hypertension (n = 3), oral pain (n = 2), thromboembolic event (n = 2) and fatigue (n = 2). Eleven patients required dose reduction due to drug related AEs [187]	

Only select studies within the past 5 years have been included due to extent of clinical trials

*AE* adverse events, *CRC* colorectal cancer, *CRPC* castrate-resistant prostate cancer, *DCR* disease control rate, *DNMT* DNA methyltransferase, *HCC* hepatocellular carcinoma, *HDAC* histone deacetylase, *HR* hormone receptor, *ITT* intention-to-treat, *NPC* nasopharyngeal carcinoma, *NSCLC* non-small cell lung cancer, *ORR* objective response rate, *OS* overall survival, *PD* progressive disease, *PFS* progression-free survival, *PR* partial response, *SAE* serious adverse event, *SCLC* small cell lung cancer, *SD* stable disease, *TEAE* treatment-emergent adverse event

To further explore the efficacy of epigenetic monotherapy, newer epigenetic agents have been investigated beyond HDAC and DNMT inhibitors, targeting more specific patient population with a narrowed spectrum epigenetic modulation. Among them, tazemetostat is the first FDA-approved epigenetic therapy in the solid tumor, epithelioid sarcoma [112]. ES is a rare soft tissue sarcoma that is characterized by the loss of expression in INI1/SNF5/SMARCB1. SMARCB1 (SWI/SNF related, matrix associated, actin dependent regulator of chromatin, subfamily b, member 1), a subunit of SWI/SNF (SWItch/Sucrose Non-Fermentable) chromatin remodeling complex, can repress EZH2 transcription [125]. The loss of INI1 function leads to elevated expression and recruitment of EZH2 to target genes, resulting in the upregulation of several oncogenic signaling pathways [126]. The accelerated approval of tazemetostat was based on the results of a single arm cohort in patients with metastatic or locally advanced ES who are not eligible for complete resection (NCT02601950). Nine out of sixty two patients with INI1-negative ES (15%) had partial response (PR) and six out of those nine patients (67%) had a duration of response lasting 6 months or longer. Tazemetostat was generally well tolerated [127] in the study.

In addition, early phase studies demonstrated BET inhibitors had clinical activities in patients with NMC. NMC is a rare and aggressive squamous cancer, which is commonly driven by the BRD4-NUT or BRD3-NUT fusion oncoprotein. A phase Ib study of birabresib (MK-8628/OTX015) was conducted in patients with NMC. Three out of ten patients (30%) with NMC had a PR with duration of response of 1.4 to 8.4 months [128]. In another phase I study of molibresib (GSK525762), out of nineteen NMC patients, four (21%) achieved either confirmed or unconfirmed PR and eight patients (42%) had stable disease as best response [129]. These results have demonstrated that targeting BRD4-NUT and BRD3-NUT with BET inhibitors resulted in strong antitumor activity in this rare patient population.

Another new epigenetic agent targeting a specific genetic defect in epigenetic pathways has been investigated. The phase III ClarIDHy trial (NCT02989857) evaluated the IDH1 inhibitor ivosidenib in 185 previously treated patients with IDH1-mutated advanced cholangiocarcinoma. Ivosidenib improved PFS from 1.4 months with placebo to 2.7 months (hazard ratio [HR] = 0.37; *P* < 0.001). Although the objective response rate was low (2.4%), clinical benefit was observed with stable disease (SD) in 50.8% of patients. Median OS was 10.8 months with ivosidenib versus 9.7 months with placebo (HR = 0.69; *P* = 0.06), including 57% of patients who crossed over from placebo group [130]. As a side note, the benefit of IDH1 inhibitors in patients with chondrosarcoma is controversial [131, 132], in part due to the different histological subtype with various disease aggressiveness and clinical outcome [133].

Summarized clinical trials investigating novel epigenetic drugs (single agent) in solid tumors are listed in Table 3.

**Table 3:** Clinical trials of newer epigenetic agents in solid tumors

Agent(s)	Cancer type(s)	Trial details	Trial identifier/status
*IDH inhibitors*			
Enasidenib (AG-221)	Advanced solid tumors, AITL	Phase I/II trial	NCT02273739
		Enrollment: 21 patients	Completed 6/2016
		Results: None available	
Ivosidenib (AG-120)	Advanced solid tumors, including cholangiocarcinoma, chondrosarcoma, and glioma	Phase I trial	NCT02073994
		Planned enrollment: 170 patients	Active, not recruiting
		Results: Ivosidenib demonstrated good oral exposure and a long half-life. Ivosidenib 500 mg once daily was an appropriate dose irrespective of intrinsic and extrinsic factors, including patient/disease characteristics and concomitant administration of weak CYP3A4 inhibitors/inducers. Persistent plasma 2-HG inhibition was observed in IDH1-mutant cholangiocarcinoma and chondrosarcoma [188]	
	Glioma	Phase I trial	NCT03343197
		Enrollment: 49 patients	Active, not recruiting
		Results: In cohort 1 (patients randomized 2:2:1 to AG-120 500 mg daily, AG-881 50 mg daily, or no treatment for 4 weeks preoperatively), AG-120 and AG-881 were CNS penetrant and lowered 2-HG compared to untreated samples. Cohort 2 is open and will evaluate AG-120 250 mg twice daily and AG-881 10 mg daily [189]	
	Advanced cholangiocarcinoma	Phase III trial	NCT02989857
		Planned enrollment: 186 patients	Active, not recruiting
		Results: Ivosidenib resulted in significant improvement in PFS and favorable OS trend versus placebo in IDH1-mutated advanced cholangiocarcinoma [130]	
*BET Inhibitors*			
AZD5153	Solid tumors, lymphomas	Phase I trial	NCT03205176
		Planned enrollment: 60 patients	Not recruiting
		Results: AZD5153 monotherapy appeared to be safe and tolerated at doses up to 30 mg once daily and 15 mg twice daily. Linear increase in PK was observed [190]	
Birabresib (OTX015, MK-8628)	Selected advanced solid tumors, including NMC, NSCLC, CRPC	Phase 1b trial	NCT02259114
		Enrollment: 47 patients	Completed 3/2017
		Results: The RP2D of birabresib was 80 mg once daily with continuous dosing. Clinical activity was observed in NMC (3 of 10 patients had PR). Birabresib has dose-proportional exposure based on PK analysis and a favorable safety profile [128]	
	Selected advanced solid tumors	Phase Ib trial	NCT02698176
		Enrollment: 13 patients	Terminated due to futility
		Summary: Dose escalation trial of MK-8628 in TNBC (1 patient), CRPC (9 patients), or NMC (3 patients)	
	GBM	Phase IIa trial	NCT02296476
		Enrollment:12 patients	Terminated due to futility
		Summary: Dose escalation and expansion cohort study to evaluate single-agent MK-8628 in recurrent GBM after failing standard front-line therapy	
BMS-986158	Selected advanced solid tumors, hematologic malignancies	Phase I/IIa trial:	NCT02419417
		Planned enrollment: 417 patients	Recruiting
		Results: N/A	
INCB054329	Advanced malignancies	Phase I/II trial	NCT02431260
		Enrollment: 69 patients	Terminated due to PK variability
		Summary: Open-label dose escalation and expansion study of INCB054329	
INCB057643	Advanced malignancies	Phase I/II trial	NCT02711137
		Enrollment: 136 patients	Terminated due to safety issues
		Summary: Open-label, dose escalation and dose expansion study of INCB057643 as monotherapy and in combination with standard-of-care agents in patients with advanced malignancies	
Molibresib (GSK525762)	NMC, other solid tumors	Phase I/II trial	NCT01587703
		Enrollment: 196 patients	Completed
		Results: RP2D was selected as 80 mg once daily. The most frequent treatment-related AEs of any grade were thrombocytopenia (51%), gastrointestinal events (22–42%), anemia (22%) and fatigue (20%). Among 19 patients with NUT carcinoma-4 achieved either confirmed or unconfirmed PR, 8 had SD as best response and 4 were progression-free for > 6 months [191]	
RO6870810	Advanced solid tumors	Phase I trial	NCT01987362
ZEN003694		Enrollment: 52 patients	Completed 10/2017
		Results: None available	
	Metastatic CRPC	Phase I trial	NCT02705469
		Enrollment: 44 patients	Completed 10/2017
		Results: None available	
*EZH2 Inhibitors*			
Tazemetostat (EPZ-6438)	Advanced solid tumors, B-cell lymphoma	Phase I trial	NCT03028103
		Planned enrollment: 28 patients	Active, not recruiting
		Results: None available	
	Advanced solid tumors, B-cell lymphomas	Phase I/II trial	NCT01897571
		Planned enrollment: 420 patients	Active, not recruiting
		Results: 64 patients [21 with B-cell non-Hodgkin lymphoma (NHL) and 43 with advanced solid tumors] received doses of tazemetostat. No treatment-related deaths occurred; 7 (11%) patients had non-treatment-related deaths (1 at 200 mg twice daily, 4 at 400 mg twice daily and 2 at 1600 mg twice daily). The RP2D was determined to be 800 mg twice daily. Durable objective responses, including CR, were observed in 8/21 (38%) patients with B-cell NHL and 2/43 (5%) patients with solid tumors. Tazemetostat showed a favorable safety profile and anti-tumor activity in patients with refractory B-cell NHL and advanced solid tumors. Phase 2 is ongoing [191)	
	Mesothelioma	Phase II trial	NCT02860286
		Enrollment: 74 patients	Completed 5/2019
		Results: Efficacy was assessed in 61 patients with deficient BRCA1 associated protein 1 (BAP1). Primary endpoint was met with 31 (51%) patients achieving disease control at 12 weeks and 15 patients sustained disease control at 24 weeks. Most frequent AEs of any grade include fatigue (32%), decreased appetite (28%), dyspnea (28%), and nausea (27%). Tazemetostat monotherapy had favorable toxicity profile and showed promising antitumor activity with confirmed responses and durable disease control in malignant mesothelioma [192]	
	INI1-negative tumors, relapsed/refractory synovial sarcoma	Phase II trial	NCT02601950
		Planned enrollment: 250 patients	Recruiting
		Results: 62 INI1-negative epithelioid sarcoma patients were enrolled and treated with tazemetostat 800 mg BID. ORR 15% (1.6% CR, 13% PR). There were 9/62 (15%) confirmed PR, with ORR 15% and DCR 26%. Median OS was 82.4 weeks. Most common AEs include fatigue (24/62; 39%), nausea (35%) and cancer pain (32%). Grade ≥ 3 TEAEs in ≥ 2 pts included anemia (6%) and decreased weight (3%). There were no drug-related deaths and a low discontinuation rate (1.7%). Tazemetostat was generally well tolerated and showed durable clinical response [127]. On January 23, 2020, FDA granted accelerated approval to tazemetostat (EZH2) for the treatment of adults and pediatric patients > 16 years old with metastatic or locally advanced epithelioid sarcoma who were not eligible for complete resection [107]	
*LSD1 Inhibitors*			
INCB059872	Relapsed or refractory Ewing sarcoma	Phase Ib trial	NCT03514407
		Planned enrollment: 21 patients	Terminated
		Results: N/A	
	Advanced malignancies	Phase I/II trial	NCT02712905
		Planned enrollment: 215 patients	Terminated
		Results: N/A	
Seclidemstat (SP-2577)	Advanced solid tumors	Phase I trial	NCT03895684
		Planned enrollment: 50 patients	Recruiting
		Results: N/A	
	Relapsed or refractory Ewing sarcoma	Phase I trial	NCT03600649
		Planned enrollment: 50 patients	Recruiting
		Results: N/A	

*AE* adverse events, *AITL* angioimmunoblastic T-cell lymphoma, *ALK* anaplastic lymphoma kinase, *AML* acute myeloid leukemia, *BET* bromodomain and extra-terminal, *CR* complete response, *CRC* colorectal cancer, *CRPC* castrate-resistant prostate cancer, *DLT* dose-limiting toxicities, *ER* estrogen receptor, *EZH2* enhancer of zeste homologue 2, *GBM* glioblastoma multiforme, *HMT* histone methyltransferase, *IDH* isocitrate dehydrogenase, *IDO-1* indoleamine 2,3-dioxygenase, *INI1* integrase interactor or INI1/SNF5/SMARCB1, *LSD1* lysine-specific demethylase 1A, *MDS* myelodysplastic syndrome, *MTD* maximum tolerated dose, *NSCLC* non-small cell lung cancer, *NMC* nuclear protein in testis (NUT) midline carcinoma, *PK/PD* pharmacokinetics/pharmacodynamics, *RP2D* recommended phase 2 dose, *SCLC* small cell lung cancer, *TNBC* triple-negative breast cancer

### Combination therapies in solid tumors

Due to the limited efficacy of epigenetic monotherapy as described previously, and the complexity of epigenetic modification in cancer, many trials are investigating combination therapies in solid tumors. Recent clinical trials include epigenetic modifier combinations as well as combinations of epigenetic agents with cytotoxic chemotherapy, hormonal therapies, and immune checkpoint inhibitors (ICIs).

#### Combination of DNMT inhibitors and HDAC inhibitors

Preclinical studies demonstrated that DNMT inhibitor enhances apoptosis in cancer cells induced by HDAC inhibitors, suggesting the potential synergism of DNMT in combination with HDAC inhibitors [134]. A phase I/II trial of azacitidine and entinostat in NSCLC yielded some promising results with durable responses [135]. This trial included heavily pre-treated patients who had received a median of three prior therapies. Clinical efficacy was observed with one complete response (CR) for 14 month duration, one PR for eight month duration, and ten patients with SD lasting at least 12 weeks. One of these patients had stable disease for 18 months and another for 14 months. The prolonged clinical benefit in certain patients in this trial prompted a correlative biomarker study to predict treatment response. The study collected and examined the promoter methylation status in circulating DNA from patient plasma collected before therapy (day 0) and after 1 cycle of therapy (day 29). Of these, ten out of 26 patients demonstrated a decrease in methylation during the first four weeks of treatment compared to their baseline. There was a higher response rate and improvement in overall survival in the patients with methylation changes (“methylation signature”–positive) compared to patients without methylation change (“methylation signature”–negative). The median OS and PFS were 10.42 months for the methylation signature-positive cohort versus 6.54 months for the methylation signature-negative (P = 0.035). This suggests a potential role of epigenetic therapy in NSCLC, and the important role of biomarkers to predict response and benefit in patients.

#### Epigenetic therapy with cytotoxic chemotherapy

Preclinical studies suggested that DNMT and HDAC inhibitors have the greatest efficacy when combined with chemotherapy in an attempt to re-sensitize cancers to the standard cytotoxic agents [136, 137]. Acquired resistance to the chemotherapy agents might be reversed when combined with DNMT and/or HDAC inhibitors, especially in ovarian cancers [138]. A phase I trial of low-dose decitabine combined with carboplatin was conducted in patients with recurrent platinum-resistant ovarian cancer. The low dose decitabine was tolerated and demonstrated biological activity in DNA hypomethylation. However, the clinical benefit was modest [139]. Another phase II randomized study compared guadecitabine in combination with carboplatin against second-line chemotherapy in patients with platinum-resistant ovarian cancer. It does not meet the primary endpoint and there is no difference in either median PFS or OS between the two groups [140, 141]. Similarly, in a phase I trial in patients with metastatic colorectal cancer who were exposed to irinotecan previously, guadecitabine in combination with irinotecan showed modest clinical activity with stable disease as the best response [142]. As a note, the challenge in epigenetic agents in combination with cytotoxic chemotherapies include the side effects of additive toxicities needing dose reduction of epigenetic agents. In addition, the chemotherapies cause G1/S cell cycle arrest, which may interfere with incorporation of hypomethylating agents into the DNA and RNA.

#### Epigenetic therapy with immune checkpoint inhibitors

ICIs have recently changed the cancer treatment landscape in many types of cancers. The combination of epigenetic agents with ICIs is an area of investigation in a variety of solid tumors [143]. In the clinical trial involving 45 patients with advanced-stage NSCLC who were treated with azacitidine and entinostat, five patients who had disease progression during the trial were subsequently enrolled in trials of anti-PD-1 therapy [135]. Three of the five patients achieved an objective response and the other two had SD for 24 weeks before disease progression. This clinical observation has led to pre-clinical research to understand the mechanism of epigenetic therapies in modulating immune responses. Treatment of tumor cells with DNMT inhibitors can induce the transcription of endogenous retrovirus (ERVs), which are normally silenced in most somatic tissues [144]. The reactivation of ERVs result in the formation of cytoplasmic double-stranded RNAs [145, 146], the cognate ligand of the retinoic acid inducible gene I (RIG-I)-like receptors (RLR), including RIG-I and melanoma differentiation associated gene 5 (MDA5) [147]. Activation of the RLR family (innate immune sensors) initiates signaling cascades leading to the production of type I and III interferons, which elicit an antitumor immune response (virial mimicry) by activation of CD8+ T cells [148, 149]. Also, epigenetic therapy can lead to the re-expression of tumor antigens, such as cancer testis antigens (CTAs) and melanoma-associated antigen 1 (MAGE1), increasing immunogenicity [150–152]. Therefore, both pre-clinical and clinical studies suggests that these epigenetic therapies might augment antitumor immune response through various mechanisms, enhancing tumor antigen expression and infiltration of cytotoxic T cells, and reversing T cell exhaustion with a concurrent increase in the abundance of effector and/or memory T cells, among others [153]. These observations are being translated into clinical trials that focus on the combination of ICIs with epigenetic drugs in a variety of solid tumors.

A phase I/Ib trial of pembrolizumab plus oral vorinostat (HDAC inhibitor) has been completed in patients with advanced/metastatic NSCLC [154]. Thirty-three patients were treated, including thirteen in phase I and twenty in phase Ib. In phase I, both ICI-naïve and ICI-pretreated patients were enrolled to determine dose-limiting toxicities (DLTs). No DLTs were observed, and the recommended phase II dose was pembrolizumab 200 mg and vorinostat 400 mg/day. The most common adverse events of any grade included fatigue (33%) and nausea/vomiting (27%). Among those 6 ICI-naïve patients, there was 1 case (16.7%) of confirmed PR, 4 cases (66.7%) of SD, and 1 case (16.7%) of PD. Of 24 ICI-pretreated patients evaluable for response, there were 3 cases with (13%) PR (1 confirmed), 11 cases with (46%) SD and 10 cases (42%) with progressive disease (PD). The results suggested the combined therapy of pembrolizumab and vorinostat is feasible with a manageable safety profile and active in both ICI-naïve and -exposed NSCLC patients. The presence of CD8+ T-cell in tumor stroma in pretreatment samples, not CD8+ T-cell in tumor bed, was associated with treatment benefit. In addition, on-treatment biopsies showed the increase in CD8+ T cells in the stroma was correlated with clinical benefit (with SD or PR for a period of ≥ 24 weeks). It would be crucial to investigate whether the combination is better than ICI alone in ICI-naïve patients in the front line setting and/or if the combination is superior to the standard of care in ICI-exposed patients in the later line treatment setting. An ongoing randomized phase 2 trial is examining pembrolizumab +/− vorinostat in ICI-naive advanced/metastatic NSCLC patients (NCT02638090).

Similarly, a phase II study is investigating azacitidine and entinostat with concurrent nivolumab in patients with metastatic NSCLC, in both ICI-naïve and ICI-resistant patient populations (NCT01928576) and a phase I study is investigating pembrolizumab in combination with guadecitabine and mocetinostat for patients with advanced lung cancer who progressed on prior ICIs (NCT03220477). These on-going trials include correlative studies to evaluate induced viral mimicry, interferon induction, and T cell function phenotypes [153].

The newer epigenetic agents in combination with ICIs are also under investigation. A phase I/II trial is evaluating a BET inhibitor, INCB057643, in combination with pembrolizumab and epacadostat (indoleamine 2, 3-dioxygenase or IDO-1 inhibitor) in patients with advanced or metastatic solid tumors (NCT02959437). Additionally, trials of EZH2 inhibitors in combination with ipilimumab (CTLA-4 inhibitor) or pembrolizumab are recruiting the patients with advanced solid tumors (NCT03525795 and NCT03854474).

#### Epigenetic therapy with other anticancer therapies

New approaches combining epigenetic agents with other anticancer therapies, including hormonal therapy, have been explored as an approach to overcome treatment resistance. In the phase II study ENCORE301, entinostat was added to exemestane (steroidal aromatase inhibitor [AI]) in patients with hormone receptor (HR)-positive advanced breast cancer with disease progression after prior non-steroidal AI. The study demonstrated a significant improvement in PFS (HR = 0.73; p = 0.06) and also in OS (HR = 0.59; p = 0.036). The combination was well tolerated, with neutropenia (13%) and fatigue (11%) being the most frequent grade 3 or 4 toxicities in entinostat-treated patients [155]. Therefore, entinostat, when added to exemestane, was designated by the FDA as breakthrough therapy for postmenopausal women with HR-positive advanced breast cancer whose disease has progressed after nonsteroidal AI therapy. Based on the ENCORE301 study, a phase III trial (E2112) is ongoing to investigate entinostat versus placebo in combination with exemestane in patients with locally advanced or metastatic breast cancer who have experienced disease progression after a non-steroidal AI [156]

Everolimus, a sirolimus (formerly called rapamycin) derivative, inhibits phosphatidylinositol 3-kinase (PI3K)/Akt/(158)mammalian target of rapamycin (mTOR) signaling pathway, which is one of the mechanisms of endocrine resistance in HR-positive breast cancer [157, 158]. In preclinical studies, the use of everolimus in combination with aromatase inhibitors results in synergistic inhibition of the proliferation and induction of apoptosis [159]. The BOLERO-2 trial showed that everolimus in combination with exemestane improved PFS compared to exemestane alone in post-menopausal women with advanced HR+/Her2-negative breast cancer [160]. However, recent data suggested that the combination of exemestane and everolimus did not yield a durable clinical response, indicating a need for alternative combinations and therapeutic strategies [161]. The pre-clinical studies showed that resistance to everolimus was mediated by overexpression of MYC in ER-positive cancers, which can be reversed by BET inhibitors [162]. Also, a combination of BET inhibitor with fulvestrant (ER degrader) showed long-lasting antitumor effect in a tamoxifen (selective ER modulator)-resistant breast cancer xenograft mouse model [163].

Similarly, the combination of BET inhibitors with AR antagonists is able to subvert resistance in castrate-resistant prostate cancer (CRPC) in preclinical experiments [164]. Other studies combining BET and PARP inhibition show mitotic catastrophe (cell death related to premature entry of cells into mitosis) with induction of apoptosis, causing synergistic effect in suppressing BRCA1/2 wild-type ovarian cancer. This study also suggests that BET inhibitors re-sensitize PARP-inhibitor-resistant BRCA mutant epithelial ovarian cancer cells to PARP inhibition [165]. DNMT inhibitors create a “BRCAness” phenotype through downregulating expression of key homologous recombination and nonhomologous end-joining (NHEJ) genes, and promote synergism with PARP inhibitors in the setting of BRCA-proficient NSCLC in animal models. These pre-clinical data support the expansion of therapeutic studies of PARP inhibitors and various epigenetic agents in patients with BRCA-proficient cancer [166].

There are also ongoing clinical trials with BET inhibitors in combination with PARP inhibitors, ER antagonists, and AR antagonists. A phase I trial is accruing patients to investigate AZD5153 in combination with olaparib for platinum-resistant/refractory ovarian cancer. Other accruing studies include a phase II trial of ZEN003694 in combination with talazoparib in TNBC (NCT03901469); a phase I/II trial to test GSK525762 in combination with fulvestrant in advanced HR-positive breast cancer (NCT02964507); and a phase Ib study combining GSK525762 with abiraterone or enzalutamide in advanced CRPC (NCT03150056). In addition, several early phase trials are investigating EZH2 inhibitors in combination with enzalutamide or abiraterone in metastatic CRPC, given the synergistic effect of EZH2 inhibitors in combination with AR antagonists.

Ongoing clinical trials of combination therapies of epigenetic drugs with chemotherapy or other agents including ICIs in solid tumors are listed in Table 4.

**Table 4:** Combination therapies of epigenetic drugs in solid tumors

Agent(s)	Cancer type(s)	Trial details	Trial identifier/status
*Combination of epigenetic agents*			
Azacitidine (DNMTi) + entinostat (HDACi)	Advanced breast cancer	Phase II trial	NCT01349959
		Enrollment: 58 patients	Active, not recruiting
		Results: Combination therapy was well tolerated but primary endpoint (ORR) was not met [193]	
Azacitidine + entinostat	Recurrent advanced NSCLC	Phase I/II	NCT00387465
		Enrollment: 94 patients	Completed 11/2014
		Results: Combined low-dose azacitidine and entinostat was well tolerated and resulted in objective, durable responses in pretreated patients with recurrent advanced NSCLC. Median survival in the entire cohort was 6.4 months [135]	
CC-486 + romidepsin (HDACi)	Advanced solid tumors, HPV + NPC, HPV + cervical cancer, liposarcoma	Phase I trial	NCT01537744
		Enrollment: 18 patients	Completed 9/2016
		Results: Although the recommended combination was tolerable, no significant anticancer activity was observed [194]	
Azacitidine + vorinostat (HDACi)	Locally recurrent and metastatic NPC and nasal natural killer T-cell lymphoma	Phase I trial	NCT00336063
		Enrollment: 18 patients	Active, not recruiting
		Results: Eleven patients were treated at 3 dose levels. This combination appeared tolerable at dose level 3 (azacitidine 25 mg/m^2^ + vorinostat 100 mg twice daily). DLTs include grade 4 thrombocytopenia, grade 3 nausea, vomiting and fatigue and grade 5 hepatic failure, and worsening of pre-existing Sweet’s Syndrome. Common grade 1/2 AEs were fatigue (73%), cough (64%), anorexia (55%) and injection site reaction (45%). One minor response was seen and 5 patients had prolonged stable disease (> 16 weeks) [195]	
*Combination with Chemotherapy or Other Agents*			
Azacitidine + capecitabine and oxaliplatin	Metastatic CRC	Phase I/II trial	NCT01193517
		Enrollment: 26 patients	Completed 11/2016
		Results: Fifteen patients in phase I and 11 in phase II were evaluable. No DLTs observed. Combination azacitidine, capecitabine and oxaliplatin was well tolerated with high rates of SD in CIMP-high patients but no objective responses seen [196]	
Azacitidine + nab-paclitaxel	Advanced or metastatic solid tumors, including HER2-negative breast cancer	Phase I/II trial	NCT00748553
		Enrollment: 30 patients	Completed 10/2015
		Results: In the phase I cohort (16 patients, with at least one prior therapy): Response rate was 61.5%. In the phase II cohort (14 patients without prior therapy): ORR 53.8% and PFS data not collected. Most common AEs were leukopenia (43.33%), nausea (36.67%), fatigue (60%) and neuropathy (46.67%) [197]	
CC-486 + nab-paclitaxel	Advanced NSCLC	Phase II trial	NCT02250326
		Enrollment: 240 patients	Active, not recruiting
		Results: Median PFS 3.2 months vs. 2.2 months, DCR 65.4% (CR/PR 13.6%) vs. 67.5% (CR/PR 16.3%) and median OS 8.1 months vs. 17.0 months for nab-paclitaxel + CC-486 arm vs. nab-paclitaxel only arm. Grade 3 or higher TEAEs occurred at 40.5% in the combination arm and 31.6% in the nab-paclitaxel alone arm. There was no survival benefit from the addition of CC-486 to nab-paclitaxel [198]	
Decitabine + temozolomide	Metastatic melanoma	Phase I/II trial	NCT00715793
		Enrollment: 39 patients	Completed 8/2015
		Results: ORR 18%, DCR 61%, median PFS 3.4 months, median OS 12.4 months and 1-year OS rate 56%. DLT was neutropenia in 6 patients. Common non-hematologic toxicities were fatigue and nausea. The combination of decitabine and temozolomide was safe and suggested possible superiority over the historical 1-year OS rate [199]	
Decitabine + tetrahydrouridine/THU-DAC	Advanced pancreatic cancer	Phase I trial	NCT02847000
		Enrollment: 13 patients	Completed 10/2017
		Results: Eight patients underwent evaluation scans at 8 weeks with SD in 1 patient and PD in 7. Common reasons for treatment discontinuation were PD (n = 6), physician discretion (n = 3) and AEs (n = 2). THU-DAC was deemed to be safe [200]	
Guadecitabine/SGI-110 (DNMTi) + carboplatin	Recurrent ovarian cancer	Phase II trial	NCT01696032
		Enrollment: 120 patients	Completed 8/2016
		Results: Overall response rate 16% in guadecitabine + carboplatin (G + C) arm versus 8% in the TC (treatment of choice) arm. The study did not meet its primary endpoint as the median PFS was not statistically different between arms (16.3 weeks vs. 9.1 weeks in the G + C and TC groups). However, the 6-month PFS rate was significantly higher in the G + C group. There was no difference between the two arms in OS [140]	
Guadecitabine + cisplatin	Refractory germ cell tumor	Phase I trial	NCT02429466
		Planned enrollment: 14 patients	Completed
		Results: MTD was guadecitabine 30 mg/m^2^ × 5 days and cisplatin 100 mg/m^2^ (with growth factor support). DLT was neutropenic fever. Most common toxicities were neutropenia (82% any grade), thrombocytopenia (42%), anemia (33%), neutropenic fever (8%) and diarrhea (8%). There were 2/14 CR lasting > 6 months, 2 PR and 1 SD. ORR 28.5%. Guadecitabine + cisplatin at MTD showed promising antitumor activity in this refractory germ cell population [201]	
Guadecitabine + irinotecan	Metastatic CRC	Phase I/II trial	NCT01896856
		Enrollment: 118 patients	Completed 8/2019
		Results: 22 patients were treated across four dose levels. DLTs were neutropenic fever, biliary drain infection, colonic obstruction and severe dehydration. Most common toxicities were neutropenia (82% any grade, 77% grade 3/4), neutropenic fever (23%), leukopenia (73% any grade, 50% grade 3/4) and injection site reactions (64% total, 0% Grade 3/4). 12/17 evaluable patients had SD as best response [202]	
Belinostat + cisplatin and etoposide	SCLC and other cancers of neuroendocrine origin	Phase I trial	NCT00926640
		Enrollment: 28 patients	Completed 4/2018
		Results: Hematologic toxicities were most common. Objective responses were seen in 11 (39%) of 28 patients; 13/28 (46%) had SD and 4 (14%) had PD. Among patients with neuroendocrine tumors, including SCLC, 7 (47%) of 15 patients achieved PR, 7 (47%) had SD and 1 (7%) had PD. There were no CR. The combination was safe, although some patients were more susceptible to AEs, and showed clinical activity in SCLC and other neuroendocrine cancers [203]	
Mocetinostat (HDACi) + gemcitabine	Metastatic leiomyosarcoma	Phase II trial	NCT02303262
		Enrollment: 20 patients	Completed 12/2016
		Results: Best responses included 1 PR and 12 SD in 18 evaluable patients. Median duration of response 2 months and median PFS 2 months. Although mocetinostat can be safely combined with gemcitabine in this population, the study could not demonstrate that mocetinostat can reverse chemoresistance in patients with previously established gemcitabine-resistant leiomyosarcoma [204]	
Panobinostat + bevacizumab	Recurrent high grade glioma	Phase I/II trial	NCT00859222
		Enrollment: 51 patients	Completed 12/2015
		Results: Although reasonably well tolerated, adding panobinostat to bevacizumab did not significantly improve 6-month PFS compared with historical controls of bevacizumab monotherapy in either cohort [205, 206]	
Vorinostat + sorafenib	Advanced HCC	Phase I trial	NCT01075113
		Enrollment: 16 patients	Completed 7/2019
		Results: Although some patients had durable disease control, the addition of vorinostat to sorafenib led to toxicities in most patients [207]	
Vorinostat + capecitabine and cisplatin	Metastatic or recurrent gastric cancer	Phase I/II trial	NCT01045538
		Enrollment: 45 patients	Completed 4/2016
		Results: ORR 42%, median PFS 5.9 months, 6-month PFS rate 44.4% and median OS 12.7 months. Did not meet primary end point (6-month PFS rate) and more AEs were observed in comparison with historical data from fluoropyrimidine–platinum doublet regimens [208]	
ZEN003694 + enzalutamide	Metastatic CRPC	Phase Ib/IIa trial	NCT02711956
		Planned enrollment: 75 patients	Completed
		Results: The most common treatment-related AEs of any grade included transient photophobia (66%), nausea (40%), fatigue (31%), decreased appetite (22%) and dysgeusia (16%). The overall median time to progression was 44.4 weeks (similar in subgroups with prior abiraterone or enzalutamide resistance) and durable responses were observed. ZEN003694 in combination with enzalutamide had acceptable toxicity profile and promising activity in metastatic CRPC refractory to enzalutamide or abiraterone [209]	
Molibresib/GSK525762 (BET inhibitor) + fulvestrant	Advanced breast cancer	Phase I/II trial	NCT02964507
		Planned enrollment: 294 patients	Active, not recruiting
		Results: N/A	
Molibresib + abiraterone or enzalutamide	CRPC	Phase Ib trial	NCT03150056
		Planned enrollment: 130 patients	Active, not recruiting
		Results: N/A	
*Combination with Immune Checkpoint Inhibitor (ICI)*			
Decitabine + durvalumab and tremelimumab	Recurrent and/or metastatic HNSCC	Phase Ib/II trial	NCT03019003
		Planned enrollment: 59 patients	Recruiting
		Results: N/A	
Azacitidine + pembrolizumab	Advanced pancreatic cancer	Phase II trial	NCT03264404
		Planned enrollment: 31 patients	Recruiting
		Results: N/A	
Azacitidine + pembrolizumab	Metastatic CRC (microsatellite stable, MSS)	Phase II trial	NCT02260440
		Enrollment: 31 patients	Completed 9/2017
		Results: ORR was 3% (1/30). Median PFS was 2.1 months and median OS was 6.2 months. Treatment-related AEs were reported in 63% of patients but most were grade 1/2 (96%). Azacitidine + pembrolizumab demonstrated tolerable safety profile but minimal antitumor activity in MSS metastatic CRC [210]	
CC-486 + pembrolizumab	Metastatic NSCLC	Phase II trial	NCT02546986
		Enrollment: 100 patients	Active, not recruiting
		Results: PFS 2.9 months versus 4.0 months, DCR 25.5% versus 38.8%, OS 11.9 months versus NA for azacitidine + pembrolizumab arm versus placebo + pembrolizumab arm. For the azacitidine + pembrolizumab arm, 49% of patients experienced any grade 3/4 TEAE related to study drug (vs. 20.4%) [211]	
CC-486 + pembrolizumab	Platinum-resistant epithelial ovarian, fallopian tube or primary peritoneal cancer	Phase II trial	NCT02900560
		Enrollment: 34 patients	Active, not recruiting
		Results: None available	
CC-486 + pembrolizumab	Metastatic melanoma	Phase II trial	NCT02816021
		Planned enrollment: 71 patients	Recruiting
		Results: N/A	
THU-DAC + pembrolizumab	Unresectable locally advanced or metastatic NSCLC and esophageal carcinomas	Phase I/II trial	NCT03233724
		Planned enrollment: 85 patients	Recruiting
		Results: N/A	
Decitabine + pembrolizumab (followed by standard neoadjuvant chemotherapy)	Locally advanced HER2-negative breast cancer	Phase II trial	NCT02957968
		Planned enrollment: 32 patients	Recruiting
		Results: N/A	
Guadecitabine + durvalumab	Advanced RCC	Phase Ib/II trial	NCT03308396
		Planned enrollment: 58 patients	Recruiting
		Results: N/A	
Guadecitabine + durvalumab and tremelimumab	Extensive-stage SCLC	Phase I trial	NCT03085849
		Enrollment: 2 patients	Completed 11/2018
		Results: None available	
Guadecitabine + durvalumab	Advanced HCC, pancreatic adenocarcinoma, cholangiocarcinoma	Phase Ib trial	NCT03257761
		Planned enrollment: 90 patients	Recruiting
		Results: N/A	
Guadecitabine + pembrolizumab	Recurrent ovarian, primary peritoneal, or fallopian tube cancer	Phase II trial	NCT02901899
		Enrollment: 35 patients	Active, not recruiting
		Results: None available	
Guadecitabine + atezolizumab	Recurrent/advanced urothelial carcinoma	Phase II trial	NCT03179943
		Planned enrollment: 53 patients	Active, not recruiting
		Results: N/A	
Entinostat + atezolizumab	Advanced TNBC	Phase Ib/II trial	NCT02708680
		Planned enrollment: 88 patients	Status unknown
		Results: None available	
Entinostat + avelumab	Advanced epithelial ovarian cancer	Phase Ib/II trial	NCT02915523
		Enrollment: 140 patients	Active, not recruiting
		Results: N/A	
Entinostat + pembrolizumab	Advanced metastatic or recurrent NSCLC, melanoma, MMR-proficient CRC	Phase Ib/II trial	NCT02437136
		Planned enrollment: 202 patients	Status unknown
		Results: 76 patients with NSCLC who progressed on prior anti-PD/PD-L1 therapy had been enrolled (72 evaluable for response). ORR 10%, which did not meet pre-specified target, but may represent clinically meaningful activity. Reponses were independent of baseline PD-L1 expression. Median duration of response was 5.3 months and median PFS 2.8 months. An additional 50% of patients achieved disease stabilization. Most patients tolerated the therapy well [212]	
Entinostat + ipilimumab and nivolumab	Metastatic or unresectable HER2-negative breast cancer	Phase I trial	NCT02453620
		Enrollment: 45 patients	Active, not recruiting
		Results: None available	
Entinostat + bevacizumab and atezolizumab	Advanced RCC	Phase I/II trial	NCT03024437
		Planned enrollment: 62 patients	Recruiting
		Results: N/A	
Entinostat + nivolumab	Unresectable or metastatic cholangiocarcinoma and pancreatic adenocarcinoma	Phase II trial	NCT03250273
		Planned enrollment: 54 patients	Recruiting
		Results: N/A	
Entinostat + nivolumab and ipilimumab	Metastatic RCC	Phase II trial	NCT03552380
		Planned enrollment: 53 patients	Active, not recruiting
		Results: N/A	
Mocetinostat (HDACi) + guadecitabine and pembrolizumab	NSCLC	Phase I/Ib trial	NCT03220477
		Planned enrollment: 40 patients	Recruiting
		Results: N/A	
Mocetinostat + ipilimumab and nivolumab	Melanoma	Phase Ib trial	NCT03565406
		Planned enrollment: 12 patients	Terminated
		Results: N/A	
Panobinostat + ipilimumab	Unresectable stage III/IV melanoma	Phase 1 trial	NCT02032810
		Enrollment: 17 patients	Active, not recruiting
		Results: Three patients had previous anti-PD1 therapy. Response rate was 12% (2 PR) with 35% SD. Median PFS 2.23 months (95% CI, 1.57—5.8) and median OS 20.97 months (95% CI, 8.97—NR). At tolerated doses, the addition of panobinostat does not appear to increase response to ipilimumab in advanced melanoma [213]	
Romidepsin + pembrolizumab ± azacitidine	Advanced MSS CRC	Phase I trial	NCT02512172
		Enrollment: 27 patients	Active, not recruiting
		Results: None available	
Vorinostat + pembrolizumab	Stage IV NSCLC	Phase I/II trial	NCT02638090
		Planned enrollment: 100 patients	Recruiting
		Results: None available	
Vorinostat + pembrolizumab	Recurrent metastatic HNSCC or salivary gland cancer	Phase I/II trial	NCT02538510
		Enrollment: 50 patients	Active, not recruiting
		Results: There were 25 patients with HNSCC (52% were p16 + oropharynx) and 25 with salivary gland cancers (SGC). Most common AEs were renal insufficiency (14%), fatigue (12%) and nausea (6%). There were 3 deaths on study. HNSCC group had 0 CR, 8 PR, and 5 SD while SGC group had 0 CR, 4 PR, and 14 SD. This combination demonstrated activity in HNSCC, with fewer responses in SGC [214]	
Vorinostat + pembrolizumab	Advanced renal or urothelial cell carcinoma	Phase I/Ib trial	NCT02619253
		Planned enrollment: 57 patients	Active, not recruiting
		Results: None available	
INCB057643 (BET inhibitor) + pembrolizumab and epacadostat (IDO1 inhibitor)	Advanced solid tumors, including stage IIIB or stage IV NSCLC, stage IV microsatellite-stable CRC, HNSCC, urothelial carcinoma, and melanoma	Phase I/II trial	NCT02959437
		Enrollment: 70 patients	Completed
		Azacitidine + pembrolizumab is assessed in group A; INCB057643 + Pembrolizumab + Epacadostat is assessed in group B; INCB059872 + Pembrolizumab + Epacadostat is assessed in group C	
		Results: None available	
Tazemetostat (EZH2 inhibitor) + pembrolizumab	Advanced urothelial carcinoma	Phase I/II trial	NCT03854474
		Planned enrollment: 30 patients	Recruiting
		Results: N/A	
INCB059872 (LSD1 inhibitor) + epacadostat and pembrolizumab	Advanced solid tumors, including stage IIIB or stage IV NSCLC, stage IV microsatellite-stable CRC, HNSCC, urothelial carcinoma, and melanoma	Phase I/II trial	NCT02959437
		Enrollment: 70 patients	Active, not recruiting
		Results: None available	

Only select studies within the past 5 years have been included due to extent of clinical trials

*AE* adverse event, *BET* bromodomain and extra-terminal, *CIMP* CpG island methylator phenotype, *CR* complete response, *CRC* colorectal cancer, *CRPC* castrate-resistant prostate cancer, *DCR* disease control rate, *DLT* dose-limiting toxicities, *DNMTi* DNA methyltransferase inhibitor, *EZH2* enhancer of zeste homologue 2, *GBM* glioblastoma multiforme, *HCC* hepatocellular carcinoma, *HDACi* histone deacetylase inhibitor, *HER2* human epidermal growth factor receptor 2, *HNSCC* head and neck squamous cell carcinoma, *HPV* human papillomavirus, *IDH* isocitrate dehydrogenase, *IDO-1* indoleamine 2,3-dioxygenase, *ITT* intention-to-treat, *LSD1* lysine-specific demethylase 1A, *MMR* mismatch-repair, *MSS* microsatellite stable, *MTD* maximum tolerated dose, *NPC* nasopharyngeal carcinoma, *NSCLC* non-small cell lung cancer, *ORR* objective response rate, *OS* overall survival, *PD* progressive disease, *PFS* progression-free survival, *PR* partial response, *RCC* renal cell carcinoma, *RP2D* recommended phase 2 dose, *SAE* serious adverse event, *SCLC* small cell lung cancer, *SD* stable disease, *SGC* salivary gland cancer, *TEAE* treatment-emergent adverse events, *TNBC* triple-negative breast cancer

## Conclusions

The development of epigenetic therapeutics has promise for cancer treatment, particularly with advancements in hematologic malignancies. In solid tumors, only one epigenetic agent (EZH2 inhibitor, tazemetostat) has been approved (ES). It is not fully understood why solid tumors are not as sensitive to epigenetic agents, even though there is profound aberrant epigenetic alterations in solid tumors. There may be a critical difference in cellular differentiation and epigenetic plasticity between solid tumors and hematological malignancies. Solid tumors arise from a more terminally differentiated state, which may be intrinsically more resistant to epigenetic reprogramming. In contrast, hematopoietic lineages are precisely controlled by epigenetic modulation. It is understandable that epigenetic agents demonstrated robust clinical activity in hematological malignancies in which cell differentiation is a key biological feature. The alternative explanation could be that altered epigenetic modulation may occur early in oncogenesis, however, it is not the “driver” event that controls the tumor cell proliferation and survival [167]. In the era of precision oncology, the broad impact of epigenetic treatment is both promising in “reprograming” solid tumor epigenetic dysfunction, as well as challenging in targeting particular epigenetic driving events. In recent years, the further development of next generation of broad spectrum agents and the emerging narrow spectrum agents as potential targeted epigenetic therapy have provided the new opportunities for solid tumor therapy. The approval of an epigenetic agent (EZH2 inhibitor, tazemetostat) in treatment of a rare soft tissue malignancy, epithelioid sarcoma, is a solid step towards the future breakthrough in the mechanism based solid tumor epigenetic treatment.

Various HDAC and DNMT inhibitors have been tested for treatment of both hematologic malignancies and solid tumors. Primary and secondary resistance to these therapies are common [168, 169]. No clear clinical benefits have been observed as yet in solid tumors. The limited antitumor activity with DNMT and HDAC inhibitors as monotherapy in solid tumors may also be related to either the short half-lives of the S phase-specific drugs with low incorporation into DNA [115] or due to a lack of specificity. Combination therapies with dual DNMT and HDAC inhibitors are explored in clinical trials; the therapeutic rationale is that densely methylated DNA is usually accompanied by deacetylated histone (transcriptionally repressive) [170]. However, most of the dual-agent epigenetic therapy trials did not result in an obvious clinical benefit, except the observation of durable responses in select NSCLC patients [135].

Potential novel therapies are being investigated to target new epigenetic modulation, such as IDH mutation inhibition and LSD1 inhibition, in both hematologic and solid malignancies. Many of these agents are targeting specific genetic defects in epigenetic pathways. Ivosidenib showed improved PFS in patients with cholangiocarcinoma harboring IDH1 mutation [130]. Pre-clinical studies suggest targeted epigenetic therapy may be effective in specific patient subsets, such as LSD1 inhibitors in the treatment for SCLC [69]. Early phase studies demonstrated BET inhibitors had activities in NMC, which is driven by BET fusion proteins. Most recently, METTL3 inhibitors and other agents targeting RNA epigenetics are emerging as potential cancer therapies with pending clinical trials.

The exciting finding that epigenetic agents are able to modulate tumor microenvironment has been a focus of epigenetic research. The combination of these “reprogramming” effects with other approved or novel therapies are being extensively explored. One of the current focuses is the combined epigenetic and immune therapy. It may be speculated that epigenetic agents have a significant “reprogramming” activity in immune cell components in addition to cancer cell component. There are many ongoing clinical trials evaluating the combination of the epigenetic agents with ICI in solid tumors. DNMT, HDAC, and other epigenetic inhibitors may enhance the response to and/or reverse the resistance to ICIs, if these agents can modulate key components of the tumor microenvironment including tumor cells, stromal cells, and innate and/or adaptive immune cells.

Beyond the scope of the current review, there are also important implications of epigenetic biomarkers in cancer screening, diagnosis, prognosis, and prediction to treatment. The development in the epigenetic biomarkers field are addressed in other reviews, including this one by Berdasco et al. [171].

In summary, epigenetic drugs represent “genomic medicines” that do not require existing DNA mutations. Given the wide diversity of solid tumors, epigenetic therapy is attractive because of the potential to target and modify the cancer genome functions. It is likely that cancer cells exploit epigenetic modulation to activate cellular pathways in cancer cell survival, including drug resistance and immune surveillance. Thus, epigenetic agents may have great therapeutic potential in the future under the right contexts. It will be essential to continue fundamental research to better identify the underlying mechanism and to translate these findings into clinical trial of newer epigenetic agents and optimize combinatorial approaches with exploration of predictive biomarkers in solid tumors.

## Data Availability

Not applicable.

## Abbreviations

2-HG: 2-Hydroxyglutarate
5caC: 5-Carboxylcytosine
5fC: 5-Formylcytosine
5hmC: 5-Hydroxymethylcytosine
5mC: 5-Methylcytosine
ALKBH5: AlkB homolog 5
AML: Acute myeloid leukemia
AR: Androgen receptor
BCL-2: B cell lymphoma 2
BET: Bromodomain and extra-terminal motif proteins
BRD: Bromodomain
c-MYC: Cellular myelocytomatosis gene
CR: Complete response
CTCL: Cutaneous T cell lymphoma
DNA: Deoxyribonucleic acid
DNMT: DNA methyltransferase
DOT1L: DOT1-like histone lysine methyltransferase
eIF3: Eukaryotic initiation factor 3
ER: Estrogen receptor
ES: Epithelioid sarcoma
EZH2: Enhancer of zeste homolog 2
FDA: U.S. Food and Drug Administration
FTO: Fat-mass and obesity associated protein
GSK525762: Molibresib
H2A: Histone 2A
H2B: Histone 2B
H3B: Histone 3B
H4: Histone 4
HAT: Histone acetyltransferases
HDAC: Histone deacetylase
HMT: Histone methyltransferases
HR: Hazard ratio
ICI: Immune checkpoint inhibitor
IDH: Isocitrate dehydrogenase
LSD1: Lysine-specific histone demethylase 1
MDS: Myelodysplastic syndrome
METTL: Methyltransferase-like protein
MK-8628/OTX015: Birabresib
MLL: Mixed-lineage lymphoma
NMC: NUT midline carcinoma
NSCLC: Non-small cell lung cancer
NUT: Nuclear protein in testis
OS: Overall survival
PR: Partial response
PRC2: Polycomb repressive complex 2
PTCL: Peripheral T cell lymphoma
P-TEFb: Positive transcription elongation factor b
R/R FL: Relapsed/refractory follicular lymphoma
RNA: Ribonucleic acid
SCLC: Small cell lung cancer
SD: Stable disease
SGI-110: Guadecitabine
SIRT: Sir-2 related
SMARCB1/INI: SWI/SNF related, matrix associated, actin dependent regulator of chromatin, subfamily b, member 1
SWI/SNF: Switch/Sucrose non-fermentable
TCA: Tricarboxylic acid
TET: Ten-eleven translocation
TP53: Tumor protein 53
YTHDF: YTH domain family
α-KG: α-Ketoglutarate
